# Efferent Control in Musicians: A Review

**DOI:** 10.3390/audiolres13010007

**Published:** 2023-01-06

**Authors:** Francisca Acuña, Rodrigo Jeria, Elisabeth Pavez, Enzo Aguilar-Vidal

**Affiliations:** 1 Escuela de Tecnología Médica, Facultad de Medicina, Universidad de Chile, Santiago 8380453, Chile; 2 Departamento de Tecnología Médica, Facultad de Medicina, Universidad de Chile, Santiago 8380453, Chile

**Keywords:** auditory efferent system, olivocochlear, musicians

## Abstract

It is widely established that musicians possess a higher level in certain auditory perceptual abilities when compared to non-musicians. This improvement may be mediated, at least in part, by changes in the cochlear response induced by reflex activation of the olivocochlear efferent system. In this review, we describe and analyze the scientific evidence regarding possible differences in the efferent response in musicians and non-musicians. The main evidence observed is that musicians present a greater robustness of the efferent olivocochlear reflex when measured by suppression of otoacoustic emissions and compared to non-musicians. Analyzing the articles presented in this review, it is possible to point out that the differential role of the efferent effect in musicians is not yet established. There is not enough evidence to support the idea that the olivocochlear system favors comparative changes in the properties of musicians’ auditory filters. New studies with psychoacoustic techniques, among others, are needed to measure the effect of the olivocochlear reflex on tuning, gain, compression, or temporal resolution in musicians and non-musicians.

## 1. Introduction

In the auditory system, there is a current of efferent information that travels from the auditory cortex to the periphery, allowing one to modulate some properties of the cochlear response through the olivocochlear system. Anatomical studies in rodents suggest that the auditory efferent system originates mainly in layers V and VI of the primary auditory cortex [1]. From here, two main pathways project first to the medial geniculate body of the thalamus and second to the other subcortical nuclei: the inferior colliculus, the cochlear nucleus, and the superior olivary complex (SOC) [2]. The olivocochlear system is formed by neuronal connections coming from the SOC that synapse mainly with the outer hair cells (OHC) and the rostral portion of the auditory nerve fibers [3]. According to the origin of the efferent fibers, two olivocochlear bundles with special and distinctive characteristics can be identified. These are the medial olivocochlear bundle (MOC) and the lateral olivocochlear bundle (LOC). The fibers of the MOC are myelinated and originate in the medial portion of the SOC. Their efferent fibers synapse directly with the base of the outer hair cells (OHCs); on the contrary, the fibers of the LOC are unmyelinated and originate in the lateral portion of the SOC, and their efferent fibers synapse directly with the type I afferent fibers of the spiral ganglion, close to the afferent synapses of these fibers with the inner hair cells’ IHCs [3]. Both fiber bundles are projected asymmetrically ipsilaterally and contralaterally. The MOC projects mostly to the contralateral cochlea (crossed), whereas the LOC projects to the ipsilateral cochlea (uncrossed) [2].

There is no evidence that the LOC can be reflexively activated by auditory stimulation; on the contrary, the MOC can be activated by auditory stimulation presented in the same ear as the measurement, in the ear opposite to the measurement, and in both ears [4,5], constituting the ipsilateral, contralateral, and bilateral olivocochlear reflex, respectively.

The olivocochlear system modulates cochlear functioning through changes in the electromotility of the OHCs, producing changes in auditory sensitivity and a decrease in cochlear amplification [6].

Over the years, several studies have assigned different functions to the olivocochlear system, highlighting the adjusting of the dynamic range of the cochlea, the reducing of the masking effect produced by noise or other tones, the controlling of the sensitivity of the cochlea (according to the subject’s state of attention), the preventing of cochlear damage produced by high-intensity sounds, and the modulating of auditory afferents during the sleep–wake cycle (widely reviewed by prof. Guinan [7] and Lopez-Poveda [8]).

There is ample evidence that subjects with musical training or professional musicians possess greater auditory perceptual skills than subjects without musical training. These abilities include pitch discrimination, rhythm, and temporal resolution [9,10,11,12,13,14,15] or speech in noise perception [16,17,18,19]. This improvement has a morpho-functional correlate, expressed in gray-matter-increased volume and the gyrification of cortical regions associated mainly with auditory and motor functions [20,21,22,23,24,25]. Additionally, through the analysis of the auditory-evoked response, improvements in neural timing and the neural representation of temporal speech patterns, at cortical and subcortical levels, have been evidenced [18,26,27,28,29,30,31,32].

As it is reasonable to suppose, a large part of the evidence generated to date is related to morpho-functional changes, mainly in the upper auditory pathway at cortical and subcortical levels, that lead to improvements in auditory perception in people with musical training [33,34]. However, we should not rule out possible changes at the level of the lower auditory pathway or the cochlea. The properties of human cochlear filters, such as tuning, compression, and gain, are nonlinear. This gives us exquisite auditory perceptual properties, such as temporal resolution, frequency dynamic range, sensitivity, and others. For example, the gain of the cochlear filter allows us to perceive the stimuli of lower intensity. This effect, added to the compression, gives us a perceptual auditory dynamic range much higher than what we would obtain with a linear system. Something similar happens with the high tuning of the filters. This favors frequency discrimination, allowing us to distinguish two tones as separate stimuli, even though they are presented simultaneously and at close frequencies. The importance of the nonlinear cochlear response on auditory perception in humans is widely discussed by Professors Oxenham and Bacon [35]. As the efferent system modulates the cochlear response [36,37,38,39,40], optimizing the MOC’s efferent feedback should improve auditory perception.

Some studies have explored the possible link between the efferent system, more specifically the robustness of the olivocochlear pathway activation response, and musical training. Those studies are discussed in this paper. The results have not been entirely consistent with each other; this could be due to the different techniques and methodologies that have been used. In this review, we describe and analyze evidence on the potential impact of musical training on the auditory efferent system, specifically on the medial olivocochlear reflex (MOCR), and highlight whether music training can be associated with increased robustness of the MOCR by promoting changes in the cochlear response.

**Figure 1:**
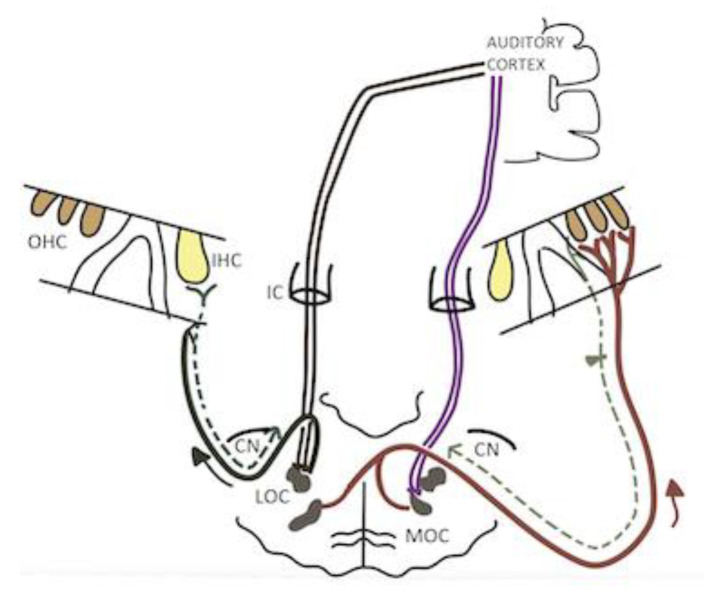
Simplified representation of the olivocochlear system.

Figure 1 represents a simplified version of the olivocochlear system. In black, the lateral olivocochlear bundle (LOC) is shown. In burgundy, is the medial olivocochlear bundle (MOC), with its crossed and uncrossed fibers. The dotted gray lines represent the afferent auditory pathways, which reach the posteroventral cochlear nuclei (CN) and cross the midline to the medial portion of the superior olivary complex. From here, the efferent neurons that will cross the midline to generate the ipsilateral medial olivocochlear reflex (MOCR) and others that will not cross generate the contralateral MOCR. In both cases, the fibers innervate the OHCs of the cochleae. Purple lines represent the descending pathway that travels from the contralateral auditory cortex to the inferior colliculus (IC) and down to connect the uncrossed MOC fibers, which will modulate the contralateral cochlear response. In brown, the descending pathway runs from the contralateral auditory cortex, through the contralateral inferior colliculus, to the LOC fibers, which modulate the activity of the auditory nerve.

## 2. Medial Olivocochlear Reflex Magnitude Measurement Techniques

To study the olivocochlear system in humans, the effect of ipsilateral and/or contralateral olivocochlear reflex activation is measured on auditory perceptual properties, and/or on cochlear response, largely employing otoacoustic emissions (OAE).

In the case of OAEs, amplitude variations are evaluated in the presence and absence of an evoked sound of the ipsilateral or contralateral MOC. The typical response is that MOCR activation decreases its amplitude. This phenomenon, known as efferent suppression of OAEs, reflects the reduction in cochlear gain. The OAEs represent an objective method to assess, at least partially, the function of IHCs.

There are several types of OAEs through which the magnitudes of the MOC response can be measured, each with different advantages and disadvantages. Transient otoacoustic emissions (TEOAE) are evoked by a broadband click or burst stimulus, are reproducible, and correlate with auditory thresholds. Distortion product otoacoustic emissions (DPOAE) are produced by the distortion generated by two simultaneous pure tones, F1 and F2 (the most robust is 2F1–F2), and they allow one to study specific frequencies independently. Stimulus-frequency otoacoustic emissions (SFOAE) are evoked by continuous pure tone and allow the evaluation of discrete frequencies. However, their origin remains controversial and it is still difficult to extrapolate their response due to the overlap between the evoked stimulus and the otoemission in the auditory meatus. These different OAE techniques have advantages and disadvantages when evaluating the magnitude of the efferent system. This was extensively reviewed by Prof. Guinan [7].

The efferent effect on hearing can also be measured using psychoacoustic techniques. For example, studying the effect of ipsi- or contralateral activation of the MOCR on the auditory filters and evidencing its effect on a slight reduction in hearing sensitivity [36,38,41] or reduction in peripheral compression and tuning [37,39,40,42,43].

## 3. Efferent Effect on Musicians

Knowing the existence of altered hearing ability in musicians [26,33], several authors have questioned whether it is not only the afferent auditory processing that improves but also the efferent effects of the auditory system. The main findings of comparative studies between musicians and non-musicians, in which the age range was from 18 to 62 years of age and there was no gender segregation, are presented in Table 1.

One of the first studying this phenomenon was Micheyl et al., 1995 [44] who proposed to study the loudness adaptation (through the tone decay test (TDT)) and the magnitude of contralateral MOCR (through contralateral suppression of TEOAEs) in musicians and non-musicians. The subjects classified as musicians were those who had been playing an instrument for more than 12 years with an average of 2 h of practice per day. To measure the suppression produced by the MOC, otoacoustic emissions were measured normally and then measured together with a contralateral 30 dB SL sound that would trigger MOC suppression. After comparing the results between musicians and non-musicians he found that the suppression produced by the MOC in musicians was greater, proposing that this was due to an efferent system, strengthened by musical training. Micheyl et al., 1997 [45], using a similar approach, compared 16 normal hearing right ears of musicians with an average age of 24 years and 10 years of musical training with 16 normal hearing right ears of non-musicians subjects with an average age of 24 years. The result was a significantly greater suppression of the magnitude of TEOAEs in musicians than in non-musicians. Subsequently, other authors [46,47] observed a greater contralateral suppressive effect on TEOAEs in normal listeners with musical training or professional musicians, respectively. Kumar et al. [48] observed a similar effect on both TEOAEs and DPOAEs in 14 rock musicians compared to 14 non-musician subjects

**Table 1:** MOCR magnitude studies in musicians (M) and non-musicians (NM).

Study	Criteria for Selection of Musicians Subjects	Age Range (Years)	Technique	Main Results
Micheyl et al., 1995 [44]	M: 12 normal hearing with musical training >12 years and 2 or more hours of daily practice (excluding percussionists) NM: 30 normal hearing	Not specified	TEOAEs suppression and loudness adaptation through the TDT	The M group showed great TEOAE suppression and less loudness adaptation than NM group
Micheyl et al., 1997 [45]	M: 16 subjects (8 females and 8 males) with at least 10 years of musical training. NM: 16 subjects (8 females and 8 males) normal hearing	M: 24.75 ± 2.86 NM: 24.06 ± 3.51	TEOAEs suppression	The M group showed great TEOAE suppression
Perrot et al., 1999 [46]	M: who began their musical training between the ages of 3 and 11 and have played an instrument an average of 4 h a day for 20 years. NM: 32 normal hearing (18 women and 14 men)	M and NM: 26.66 ± 3.74	TEOAEs suppression	The M group showed great TEOAE suppression in both right and left ear
Bidelman et al., 2017 [49]	M: 12 professional musicians (8 women and 4 men) with at least 9 years of experience. NM: 8 normal hearing subjects (3 women and 5 men)	M: 23.0 ± 4.1 NM: 23.3 ± 2.5	Contralateral DPOAEs suppression and ipsilateral DPOAEs post-onset adaptation.	The M group showed great contralateral DPOAEs suppression and stronger ipsilateral DPOAE adaptation
Kumar et al., 2016 [48]	M: 14 rock musicians, with >5 years of musical experience after the age of 10. With weekly musical practice equal to or greater than 15 h. NM: 14 Normal hearing subjects	M and NM: 18–25	TEOAEs and DPOAE suppression	The M group shows greater suppression in the magnitude of TEOAEs and DPOAEs than the NM group.
Bulut et al., 2019 [47]	M: 26 musicians of the Balkan Symphony Orchestra with at least 5 years of experience. NM: 17 normal hearing.	M: 34.3 ± 1.4 NM: 37.7 ± 4.8	TEOAEs suppression	The M group showed great TEOAE suppression
Braeshers et al., 2003 [50]	M: 29 professional musicians (17 women and 12 men) from the Louisiana Philharmonic Orchestra who had a weekly practice of 16–30 h. NM: 28 normal hearing	M: 24–61 NM: 25.4–62.8	Binaural TEOAEs suppression	The M group subjects reached higher suppression values than in the NM group.
Tarnowska et al., 2020 [51]	M: 12 in musicians (10 women and 2 men) NM: 12 normal hearing	M: 24.4 ± 1.7 NM: 24.6 ± 3.4	Measured PTCs at 2 and 4 kHz frequencies with contralateral pink noise of 60 dB SPL	PTCs were quantified using Q10; no significant difference was found between musicians and non-musicians.

Summary table of reviewed studies sorted by year of publication: Glossary: TEOAEs: Transient otoacoustic emissions, DPOAEs: Distortion product otoacoustic emissions M: Musicians, NM: Non-musicians, PTCs: Physiological tuning curves, TDT: Tone decay test.

Brashears et al. [50] measured the binaural suppression of TEOAEs in normal-hearing musicians and non-musicians (aiming to stimulate a larger number of fibers). The musician subjects (29) were members of the Louisiana Philharmonic Orchestra and had a minimum of 22 years of musical experience with an average personal musical practice of 12 h per week; on the other hand, the non-musicians had no formal training or musical experience in the last 7 years. The suppression of TEOAEs was measured with a linear click stimulus at 80 dB SPL and a broadband binaural noise as a suppressive stimulus at 70 dB SPL. An overall significant difference between the two groups is evident; moreover, the right ear values in the musicians’ group reached higher suppression values in the later time bands. The authors attribute this difference to a strengthening of the MOC response in musicians to have greater cochlear protection.

Aiming to evaluate the effect of musical training, Bidelman et al., 2017 [49] investigated the difference in MOCR in musical and non-musical subjects, evaluating the suppressive effect on DPOAEs. The authors focused their research on the hypothesis that long-term musical training can strengthen the temporal dynamics of ipsilateral and contralateral MOC feedback to the ear. To test this, 12 classically trained musicians (with at least 9 years of formal musical experience) and 8 non-musicians were evaluated. The main results show that musicians present a greater efferent effect intensity, both ipsi- and contralaterally. Interestingly, the authors found a significant relationship (*r* = 0.51) between the magnitude of the efferent effect and the level of training in years (see Figure 5B in the cited article).

A qualitatively opposite effect was observed by Stuart and Daughtrey, 2016 [52] who did not observe any difference in the magnitude of contralateral suppression of TEOAEs. In this study, the groups were differentiated according to a self-report in which the subject had to answer the question “do you consider yourself a musician?”, with a possible answer of “yes” or “no”. They also did not observe a relationship between musical perceptual skills (measured using the PROMS shortened test) and the magnitude of the MOCR. This result goes in the opposite direction of previous reports. This inconsistency could be due to the characteristics of the sample and the self-reporting criteria used to categorize the participants. In fact, in the previously mentioned studies, the criterion for being considered a musician is to have formal music studies and/or professional dedication for a significant number of years. As stated by the authors, in this study, only a small proportion of subjects meet these requirements.

As previously mentioned, the efferent effect can be measured perceptually. Unfortunately, only a small number of studies have used this approach to investigate the efferent effect, and, furthermore, there is only one study that used this approach within the purview of this review. Tarnowska et al., 2020 [51] measured the effect of MOCR on physiological tuning curves (PTCs). Using simultaneous masking, the PTCs were measured and compared between 12 musicians and 12 non-musicians, at 2 kHz and 4 kHz frequencies, with and without contralateral pink noise stimulation at 60 dB SPL. The contralateral noise decreases the filter tuning (widens the filters) and no differential effect is observed between the groups. That is, the efferent effect on tuning did not differ between musicians and non-musicians.

## 4. Discussion

### 4.1. The Strength of the Efferent Reflex

Analyzing the studies presented in this review, it is important to point out that most studies have observed a greater magnitude of efferent effect, both ipsi- and contralaterally, in musical subjects when compared to a matched group of non-musicians. Only two studies are reported in which a favorable and significant difference in favor of musicians is not observed [51,52]. In the first case, it is highlighted that the musicians and the non-musicians group were not properly differentiated according to their musical experience, but it was according to a self-report and the application of the PROMS shortened test that this could have influenced the results as there was musical heterogeneity in the groups (musicians within the NM group and non-musicians within the M group). In the second case, the authors did not measure the magnitude of the suppressor effect but instead measured the effect of efferent control on the tuning of the auditory filters (using the PTC technique). As the authors point out, the efferent effect is often small and may even go unnoticed when assessed indirectly, such as through variations in the properties of auditory filters.

### 4.2. The Implications of the Strength of MOCR

Since there is consensus on the greater robustness of the efferent reflex response in musicians, it is interesting to discuss the differential role of this effect on this group.

Most authors agree that musical training would promote greater involvement of the MOCR in the cochlear response to sounds, since musicians, generally exposed to a large number of sounds for several hours, need a superior auditory capacity that allows them to discriminate sounds very accurately, both in their frequency and temporal spectrum. A robust MOCR would imply a greater ability to discriminate and pay attention to sounds in noisy environments. Naturally, this perceptual benefit would be mediated by changes in the nonlinear properties of the cochlear response caused by the activation of the olivocochlear system.

Unfortunately, the idea outlined above is far from being evidenced to date. The greater suppressive effect of otoacoustic emissions observed in musicians is probably a manifestation of cochlear gain reduction. The perceptual manifestation of which would be a slight reduction in hearing sensitivity [36,37,38,49]. However, we do not know of any differential effect of the efferent system on, for example, tuning, compression, and time resolution in musicians. This could be due to the fact that many of the perceptual techniques that allow the inferring of the properties of auditory filters have some important complexities, which makes it difficult to implement experiments with this approach. In this regard, it highlights the long duration of some of these tests.

Only one study has evaluated the differential effect in musicians of MOCR on auditory filters (tuning) without finding differences between musician and non-musician groups, as previously noted.

Another aspect for which a MOCR would have implications would be cochlear protection. This is a plausible idea, since physiological studies in mammals have shown the protective role of the olivocochlear system against damage to the sensory and nerve cells of the first portion of the auditory pathway [53,54], where this attenuating effect on cochlear gain would be key to trauma protection. Given that musicians are often exposed to moderate- and high-intensity sounds, it is reasonable to think that another component that could contribute to generating greater robustness to MOCR is the protection against acoustic trauma. Otsuka et al., 2016 [55] conducted their research trying to determine whether the measurement of MOCR could be a reliable parameter when assessing the risk of hearing loss in musicians (if musicians have a strengthened MOCR, the variation would be greater). To evaluate temporary hearing impairment after a 1 h musical practice session, they studied 16 university violin students, quantifying the values in an audiogram and evaluating the click-evoked otoacoustic emissions (CEOAEs) before and after the musical practice. The most remarkable result is that, in the case of the left ear, which is the ear most exposed by violinists, the magnitude of the efferent reflex was negatively correlated with the magnitude of TTS change and the reduction in CEOAE post-exposure. In other words, this means that subjects with a more robust MOCR were impacted less by noise exposure, as measured by TTS and CEOAE variation.

There are some factors that could make it difficult to interpret the findings in the studies, either in the comparison of the groups in each study or in comparative analysis between the different studies. In this sense, the level of exposure to noise to which musicians are usually subjected stands out. This risk agent, which is a variable that depends on various exposure factors, such as type of instrument or number of hours of exposure [56] could cause hearing damage and, therefore, alter the pairing between groups. This is why all the studies presented here have used the inclusion criterion of being a normal listener (varying the maximum audiometric threshold criterion allowed for a given frequency between 25 d and 15 dB HL). Unfortunately, the criterion of normal hearing may not be sufficient due to the possible presence of subclinical hearing damage associated with noise exposure in musicians [57]. This is why future research should aim to test the level of noise exposure by direct measurements and/or by questionnaire/interview. In this sense, it is relevant to quantify the cumulative noise exposure, regardless of the context in which the risk exposure is provoked. Other occupational or recreational noises could cause clinical or subclinical hearing damage. Some instruments, such as The Noise Exposure Structured Interview (NESI) [58], provide an approximation of the level of exposure to potentially harmful noise throughout life and could be used in approaches similar to those analyzed here. The age of the participants could be another factor hindering the analysis. Nevertheless, the studies are age-matched and tend to concentrate on ages between 18 and 26 years. Some, such as Braeshers et al. (2003), work with an extended age range (24–62 years old) [50].

It is widely established that musical training induces brain plasticity [34,59]. Among the mechanisms involved are, at least, a cortico-leakage gradient that induces plasticity and the olivocochlear efferent loop mechanism that is strengthened by training, always mediated by motor activity and attention [60].

There is still debate about whether this greater plasticity would be produced by musical training or if they are simply people with strong perceptual skills that favor performance in musical tasks. To better elucidate this paradigm, it would be useful to have studies that evaluate the magnitude of the MOCR response and the auditory abilities of musicians when they begin to practice an instrument and to study how they evolve. If the magnitudes of the MOCR is strengthened by musical training, having periodic musical training from childhood would be highly beneficial to promote brain plasticity and ears with better auditory abilities and protect against noise exposure.

### 4.3. Methodological Considerations

A critical point in interpreting these studies is the definition of “musician”. In the studies considered, it was observed that there is no consensus on the fundamental aspects for categorizing a subject as a musician or musically trained. The diversity is as great as 5 [47] to 12 [44] years as the minimum time of musical experience. It should be noted that, in all the research cited here, the subjects were musicians or classical music students, except for one study [48], which conducted their investigations with rock musicians. It would be interesting to compare whether the type of music influences the magnitude of MOC suppression, for example, to be able to evaluate musical subjects who play classical music versus musical subjects who play rock music.

Another important aspect to consider when analyzing these studies is the approach and technique used. It should be noted that the characteristics of the evoked noise directly affect the magnitude of the efferent effect. Thus, the bandwidth or intensity of the evoked noise is directly related to the magnitude of the efferent effect [61,62,63]. This is why the studies analyzed here use broadband noise. Intensity is also a critical point, since a noise must be selected at an intensity that evokes the MOC reflex without triggering the middle ear reflex (MEM) [37,64]. Typically, the evoked noise has been around 60 dB SPL.

In most studies, TEOAEs were used to compare the magnitude of the MOC effect, which has proven to be an easy technique to perform and gives reliable results. In contrast, DPOAEs correspond to the sound pressure level (SPL) measured at the ear canal, reflecting a complex constructive and/or destructive interaction of sounds generated in the cochleae. Eventually, the efferent system may act in different magnitudes in one of the primary regions and/or out of phase, finally, generating changes in the SPL measured in the ear canal that are difficult to predict and interpret.

SFOAEs are a more recent alternative for assessing the extent of MOCR. These OAEs are comparatively the most complex to measure because the evoking tone and the OAE share frequency so they can overlap in the ear canal. This makes it very difficult to extract the magnitude of the suppressive effect. Lilaonitkul and Guinan [64,65] propose to measure SFOAEs with and without efferent activation and to quantify the magnitude of the effect as a change in OAE amplitude and phase. This would align the measurement more closely with TEOAEs, with the advantage of being able to evaluate by the frequency-specific effect.

## 5. Conclusions

Subjects with musical training have a more robust olivocochlear reflex when compared with non-musical subjects. The results show a clear trend when OAE suppression was used to assess the magnitude of the MOCR response. This phenomenon may be associated with greater control of cochlear gain by the MOCR in subjects with musical training. However, it is not established that this increased robustness of the efferent effect in musicians leads to changes in the nonlinear properties of hearing filters, such as tuning or compression.

Future studies are needed to clarify whether the olivocochlear system participates, at least in part, in favoring rich perceptual auditory properties in musicians. In this sense, it could be useful to use psychoacoustic techniques to assess the efferent effect on sensitivity, compression, tuning, or temporal resolution in subjects with and without musical training.

## Data Availability

Not applicable.
